# Differential haplotype expression in class I MHC genes during SARS-CoV-2 infection of human lung cell lines

**DOI:** 10.3389/fimmu.2022.1101526

**Published:** 2023-02-01

**Authors:** Ronaldo da Silva Francisco Junior, Jairo R. Temerozo, Cristina dos Santos Ferreira, Yasmmin Martins, Thiago Moreno L. Souza, Enrique Medina-Acosta, Ana Tereza Ribeiro de Vasconcelos

**Affiliations:** ^1^ Bioinformatics Laboratory (LABINFO), National Laboratory of Scientific Computation (LNCC/MCTIC), Petrópolis, Brazil; ^2^ Laboratory on Thymus Research, Oswaldo Cruz Institute (Fiocruz), Rio de Janeiro, Brazil; ^3^ National Institute of Science and Technology on Neuroimmunomodulation, Rio de Janeiro, Brazil; ^4^ Instituto de Cálculo, Facultad de Ciencias Exactas y Naturales, Universidad de Buenos Aires (FCEyN-UBA), Buenos Aires, Argentina; ^5^ Laboratory of Immunopharmacology, Oswaldo Cruz Institute (IOC), Oswaldo Cruz Foundation (Fiocruz), Rio de Janeiro, Brazil; ^6^ Center for Technological Development in Health (CDTS), National Institute for Science and Technology on Innovation on Neglected Diseases Neglected Populations (INCT/IDNP), Oswaldo Cruz Foundation (Fiocruz), Rio de Janeiro, Brazil; ^7^ Molecular Identification and Diagnostics Unit (NUDIM), Laboratory of Biotechnology, Center for Biosciences and Biotechnology, Universidade Estadual do Norte Fluminense Darcy Ribeiro (UENF), Campos dos Goytacazes, Brazil

**Keywords:** allele-specific expression, allele swapping, COVID-19, haplotype expression, HLA alleles, RNA-Seq, SARS-CoV-2

## Abstract

**Introduction:**

Cell entry of SARS-CoV-2 causes genome-wide disruption of the transcriptional profiles of genes and biological pathways involved in the pathogenesis of COVID-19. Expression allelic imbalance is characterized by a deviation from the Mendelian expected 1:1 expression ratio and is an important source of allele-specific heterogeneity. Expression allelic imbalance can be measured by allele-specific expression analysis (ASE) across heterozygous informative expressed single nucleotide variants (eSNVs). ASE reflects many regulatory biological phenomena that can be assessed by combining genome and transcriptome information. ASE contributes to the interindividual variability associated with the disease. We aim to estimate the transcriptome-wide impact of SARS-CoV-2 infection by analyzing eSNVs.

**Methods:**

We compared ASE profiles in the human lung cell lines Calu-3, A459, and H522 before and after infection with SARS-CoV-2 using RNA-Seq experiments.

**Results:**

We identified 34 differential ASE (DASE) sites in 13 genes (*HLA-A*, *HLA-B*, *HLA-C*, *BRD2*, *EHD2*, *GFM2*, *GSPT1*, *HAVCR1*, *MAT2A*, *NQO2*, *SUPT6H*, *TNFRSF11A*, *UMPS)*, all of which are enriched in protein binding functions and play a role in COVID-19. Most DASE sites were assigned to the MHC class I locus and were predominantly upregulated upon infection. DASE sites in the MHC class I locus also occur in iPSC-derived airway epithelium basal cells infected with SARS-CoV-2. Using an RNA-Seq haplotype reconstruction approach, we found DASE sites and adjacent eSNVs in phase (i.e., predicted on the same DNA strand), demonstrating differential haplotype expression upon infection. We found a bias towards the expression of the HLA alleles with a higher binding affinity to SARS-CoV-2 epitopes.

**Discussion:**

Independent of gene expression compensation, SARS-CoV-2 infection of human lung cell lines induces transcriptional allelic switching at the MHC loci. This suggests a response mechanism to SARS-CoV-2 infection that swaps HLA alleles with poor epitope binding affinity, an expectation supported by publicly available proteome data.

## Introduction

The coronavirus disease 2019 (COVID-19) pandemic significantly continues to burden public health response and management, with over 631 million infected people and over 6.5 million cumulative deaths worldwide (https://covid19.who.int/). The severe acute respiratory syndrome coronavirus 2 (SARS-CoV-2) infection causes from asymptomatic to life-threatening pulmonary illness with multiorgan dysfunction (1, 2). About 0.1 to 0.9% of infected people develop fatal disease outcomes (3). Epidemiological studies showed that advanced age, male biological sex, and comorbidities are major risk factors for life-threatening COVID-19 (4). Respiratory tract epithelial cells and pneumocytes are the first target cells of SARS-CoV-2. The virus enters cells by binding its Spike protein to the host angiotensin-converting enzyme 2 (ACE2) membrane receptor (5). The kinetics of the SARS-CoV-2 replicative cycle during the acute phase of infection can lead to endothelial barrier disruption, dysfunctional alveolar-capillary oxygen transmission, and impairment in oxygen diffusion capacity (6). These phenotypes are characteristic of acute respiratory distress syndrome (ARDS) and affected individuals usually demand oxygen support.

A hallmark of severe COVID-19 is the overactivation of the inflammatory response through maladaptive proinflammatory cytokine production by transendothelial leukocyte migration. The cytokine storm causes local cell damage in the alveoli and systemic inflammation. Excessive inflammation, hypoxia, immobilization, and diffuse intravascular coagulation are not uncommonly observed in COVID-19 patients. Those conditions may predispose to venous and arterial thromboembolism, ischemic stroke, and myocardial infarction, which are life-threatening complications (7). Also, SARS-CoV-2 interferes with how antigens are presented, how alveolar macrophages work, and how type I interferon works (8, 9).

Understanding the perturbations associated with SARS-CoV-2 infection in the respiratory tract cells is challenging because of the difficulty in obtaining relevant biological samples from affected subjects. To do this, *in vitro* culture models permissive to SARS-CoV-2 infection are used to investigate the underlying mechanisms of infection and disease pathology. Calu-3 and A549 culture models have been a mainstay of respiratory research in the last four decades (10, 11). Even though both cell lines are epithelial and come from adult lung non-small cell adenocarcinoma, Calu-3 is highly permissive to SARS-CoV-2 infection and replication in an ACE2-dependent way, whereas A549 is not permissive to SARS-CoV-2 due to its low expression of the ACE2 receptor (12). Notably, the exogenous expression of ACE2 in A549 renders a chemokine signature similar to that of Calu-3 cells (12). ACE2 receptor-independent models, such as the H522 lung adenocarcinoma cell line, showed that viral infection uses an alternative receptor and depends on surface heparan sulfates (13). In addition, airway epithelium basal cells (iBCs) experimentally derived from induced pluripotent stem cells (iPSCs) also reproduced the transcriptome profile of the primary human airway epithelial cells and other airway cell types (14). Comprehensive transcriptome studies with these cell lines showed genome-wide activation of genes related to type I and III IFN production, chemokine expression, NLRP3 inflammasome, metabolic hormone process, and the low-density granulocyte (LDG) gene signature (12, 15–17). Nevertheless, the impact of SARS-CoV-2 infection on allele-specific expression has not been fully explored.

Genome-wide association studies (GWAS) found that common SNVs at 17 different loci were linked to severe COVID-19 outcomes (18–20). Loss-of-function rare SNVs in genes related to inborn errors of type I IFN immunity were found in at least 3.5% of patients with pneumonia (3, 21). Variants in the Human Leukocyte Antigen (HLA) locus appear to play a role in asymptomatic and mild diseases. The highly variable HLA locus codes for proteins that activate T-cells and help the immune system fight off different pathogens. Class I and II HLA molecules present antigens to CD8+ and CD4+ T-cells, respectively. In couples discordant for COVID-19, HLA-A variants associated with symptomatic versus asymptomatic SARS-CoV-2 infection in highly exposed individuals (22). Moreover, the number of missense variants in the *MUC22* gene was higher in resilient super elders (people over 90 years old who were infected but had mild or no symptoms) (23).

Most markers found by GWAS are single-nucleotide variations (SNVs) at noncoding sites that often act as cis-regulatory variants. Expression quantitative trait loci (eQTL) analysis (24, 25) is often used to identify causal regulatory variants from GWAS, which also requires many samples besides being deeply influenced by interindividual differences (26). SARS-CoV-2 infection is known to promote imbalances in the expression of genetic variants across the human genome (27). But it still needs to be determined what their functional effects are because figuring out the links between genotype and phenotype in people with different genetic backgrounds requires analyzing many transcriptomes. For these reasons, allele-specific expression (ASE) has become the most effective assay for quantifying gene variant expression (28).

ASE analysis measures the steady-state imbalance between the transcription of the two parental alleles at heterozygous sites of the diploid genome (29). Each genetic variation is expected to show a 1:1 allelic expression ratio. The deviation from this assumption captures a dynamic regulation of biological processes related to the effects of cis-regulatory variants, genomic imprinting, X chromosome inactivation (XCI), A-to-I(G) RNA editing, nonsense-mediated decay, random monoallelic expression, or allelic exclusion (30). ASE analysis also enables the identification of gene-by-environment (GxE) interactions, highlighting the environment’s contributions to modulating the genetic effects of relevant complex traits (31). Unlike GWAS and eQTL analyses, ASE analysis quantifies the difference in the abundance of alleles in the same individual by controlling the impact of genetic background and environmental changes in replicate samples (26). Comparisons across allelic expression profiles can highlight genes potentially involved in mechanisms associated with disease. For example, Goovaerts et al., found that the parent-of-origin-dependent monoallelic expression of imprinted genes is deregulated in breast cancer (32). Pervasive perturbations in ASE sites were found in monozygotic twins discordant for Down syndrome, suggesting genome-wide dysregulation in cells with extranumerary chromosome 21 (30). Here, we describe a new way to use RNA-Seq experiments on human cell lines infected with SARS-CoV-2 to find allele-specific changes significant for COVID-19 disease.

## Materials and methods

### Biological data and sample information

We chose transcriptome studies from bulk RNA-Seq data of SARS-CoV-2 infected lung human cell lines publicly available at the Sequence Read Archive platform (**Table S1**). Only experiments comparing mock-treated and SARS-CoV-2 infected cells with two or more replicates per condition were selected. We included three different lung cell lines in our analysis: Calu-3, A549, and H522. These cell lines originated from the lung adenocarcinoma epithelium of Caucasian adult male subjects. Both A549 and H522 are ACE2-negative models supporting SARS-CoV-2 replication *via* independent entries. In our analysis, we also used A549 with an exogenous expression of ACE2. In the study by Blanco Melo et al., 2020 (GEO BioProject PRJNA615032), we selected four experiments using the A549 cell line and one from Calu-3 (12). In the study by Wyler et al. (GEO BioProject PRJNA625518), we included a longitudinal experiment of RNA-Seq in Calu-3 cells at three different time points (16). We also used RNA-Seq data of Calu-3 cells from the study by Kim D et al., 2021 (GEO BioProject PRJNA661467) at eight different time points (17). The H522 experiments were retrieved from the meta-analysis conducted by Puray-Chavez et al. (GEO BioProject PRJNA686659), which compares the transcriptional profile for four ratios of the multiplicity of infection (MOI) at six-time points (13). We also use whole-exome sequencing (WES) data for the cell lines listed above (**Table S1**) to figure out the zygotic profile of each RNA-Seq variant. Lastly, we used RNA-Seq data of airway epithelium basal cells (iBCs) made from induced pluripotent stem cells (iPSCs) (GEO BioProject PRJNA805095) to confirm what we found in the three models we used in our analysis. The human airway epithelial cells were differentiated from the BU3 NGPT and 1566 iPSC cell lines (14).

### Data processing and identification of differential allele-specific expression sites

We extracted the *fasta* files of each replicate using the *fastq-dump* function from the *sra-toolkit* (https://github.com/ncbi/sra-tools). Bioinformatic analysis was done separately for each replicate. Allelic imbalance analysis at expressed SNVs (eSNVs) sites was performed using *PipASE*, a pipeline to identify ASE sites in transcriptome data (30). We first examined the sequencing quality parameters for each *fastq* file using *fastqc* (https://www.bioinformatics.babraham.ac.uk/projects/fastqc/). Next, bad-formed reads were removed using *Trimmomatic* (33). We aligned the filtered reads to the human GRCh38 reference genome assembly with STAR v3.7 software (34). Mapped sequences were further post-processed using *SAMtools* to sort, index, and select reads based on mapping quality parameters (MAPQ ≥ 30) in *BAM* files (35). Then, we masked duplicate reads and performed variant calling in RNA-seq data using *MarkedDuplicates* and *HaplotypeCaller* from GATK v4.1, respectively (36, 37). We used *ASEReadCounter* to determine the read counts for reference and alternative alleles in each position (29). The genomic information for each variant was annotated with the help of the Ensembl Variant Effect Predictor (https://www.ensembl.org/Tools/VEP).

To estimate the impact of SARS-CoV-2 infection on the differential expression of genetic variants across the human genome, we calculated the reference allele ratio (ref ratio) in each replicate using the following equation: ref ratio = (# of reads with the reference allele)/(# of reads with the reference allele + # of reads with the alternative allele). For differential ASE analysis, we required coverage of at least ten reads per variant site and the occurrence of each site in at least two replicates in each assay condition. We used a binomial model from the stats package R (38) for differential ASE analysis at each eSNV site. Adjusted P-values for multiple comparisons were performed using the *p.adjust* function in R with the Benjamini and Hochberg method. To estimate the magnitude of the expression changes, we calculated the log2 fold change of the ASE (LogASE) for each site using *DESeq2* (39) according to the framework available by Love (2017) (40). Positive LogASE values represent the increase of the alternative allele over the reference. In contrast, negative values represent ASE sites that exhibited a preferential expression of the reference allele after infection. Only the SNVs that exhibited FDR < 0.1 and -0.95 < LogASE > 0.95 were considered differentially expressed across the conditions. We used the R package *clusterProfiler* to perform functional enrichment analysis on the set of genes that displayed differential allele expression (41). Annotations were made for Gene Ontology (GO) terms in three different areas: molecular function (MF), biological process (BP), and cellular component (CC). We performed a GO over-representation test, keeping only enriched terms that showed *p.adjust* < 5%. We also used *clusterProfiler* to do a KEGG over-representation analysis to learn more about the main metabolic pathways enriched for genes with ASE sites. Similar analyses were also performed using *ReactomePA* in R (42).

### Detection of chromosomal aberrations and haplotype inference using allelic imbalance from RNA-seq dataset

Since the tumor cell lines used in this study are hypotriploid (43), we used eSNP-Karyotyping to look for chromosomal changes in the RNA-Seq data (44). We sought to compare the karyotype of mock-treated and SARS-CoV-2-infected cells to determine whether the allelic imbalance was either generated by chromosomal differences between both samples or associated with the infection. Thus, *BAM* files from different replicates within the same condition were merged with *SAMtools* (35) and edited using *AddOrReplaceReadGroups* from Picard (https://broadinstitute.github.io/picard/) to assign a single new read-group for all the reads in the *BAM* file. The *BAM* file generated by this step was indexed with the SAMtools index, followed by a second variant call with HaplotypeCaller from GATK v4.1. We filtered out eSNVs with low coverage (below 20 reads) and low minor allele frequency (lower than 0.2). Using a window of 151 eSNVs, we estimate the moving medians of the major-to-minor allele ratios across the genomic coordinates. eSNP-Karyotyping also shows FDR-corrected P values for regions significantly altered within each sample. Combined *BAM* and *VCF* files were also used to phase eSNVs within haplotype blocks. We used a Bayesian haplotype reconstruction framework from HapTree-X to assess phased haplotype blocks from the allelic imbalance observed in RNA-Seq data (45). We passed the human *GTF* file from the Ensembl GRCh38.105 version *via* the -g parameter to improve the phasing quality.

### Sequence-based HLA typing using RNA-seq data

After the haplotype reconstruction approach, we conducted HLA allele identification directly from RNA-Seq reads in each sequence. First, RNA-Seq reads in *fastq* format were mapped to human chromosome 6 (GRCh38) using *bowtie2* (46). The mapped sequences were assembled into 200 bp contigs using the *TASR* tool (47) and aligned to HLA reference sequences using the NCBI BLAST+ 2.13.0 package (https://blast.ncbi.nlm.nih.gov/Blast.cgi). The following alignment parameters were used: -b 5 -v 5. The HLA reference sequences of classes I and II genotypes were retrieved in *fasta* format from the IMGT/HLA database. After alignment, the selected sequences were used to predict HLA alleles in the HLAminer tool with the default parameters (48). Next, the definition of HLA alleles for each sample was based on the intersection of alleles present across the different replicates of the experiments. Finally, we queried DASE sites and co-localized eSNVs affected in samples predicted to be heterozygous to verify the HLA allele preferentially expressed during SARS-CoV-2 infection.

## Results

### Allelic expression of eSNVs in the MHC class I locus is preferentially impacted in lung epithelial cell lines during SARS-CoV-2 infection

**Figure 1:**
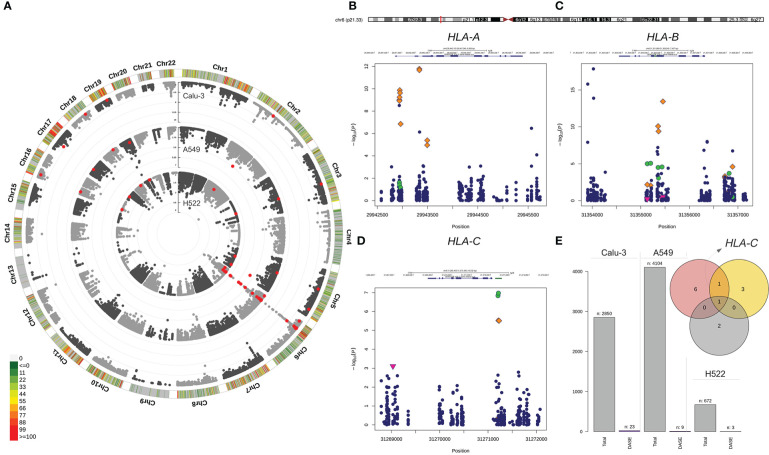
Differential allele-specific expression sites across the single-nucleotide variants identified in Calu-3, A549, and H522 lung cell lines. **(A)** Circular Manhattan plot of the chromosomal distribution of eSNVs tested using a binomial approach. The densities of eSNVs per chromosome in the Calu-3, A549, and H522 cell lines are depicted inward. Red points represent DASE sites with FDR < 10%. **(B–D)** Regional plot of classical MHC class I genes with the orange diamond showing the DASE sites in Calu-3, green circles for A549, and H522 represented by the pink triangle point down. **(E)** The total number of eSNV sites tested in each lung cell line, followed by the number of DASE sites found. The intersection between the genes harboring DASE sites in the three cell lines is depicted in the Venn diagram. *HLA-C* was the only gene that showed DASE sites in all lines. Nevertheless, *HLA-B* was also shared between Calu-3 (red circle) and A549 (yellow circle).

We compared the allelic expression profiles of eSNVs in bulk RNA-Seq data from Calu-3, A549, and H522 lung cell lines before and after SARS-CoV-2 infection. We interrogated 6,884 heterozygous eSNVs detected across the mock-treated and SARS-CoV-2-infected comparisons, with coverage ≥ 35 reads at each site. Thirty-four eSNVs displayed differential allele-specific expression (DASE) after viral infection (**Figure 1**; **Table S2**). These sites were heterozygous in the WES data of their respective cell lines. The ACE2-dependent Calu-3 model, harbored 68% (n=23/2,50) of all DASE sites. We also noticed seven eSNVs significantly altered in A549 with exogenous expression of ACE2 (n = 7/4,094). The ACE2-independent models of H522 and A549 showed the smallest DASE sites with four (n = 4/672) and two eSNVs (n = 2/872), respectively (**Figure 1**). The read depth at DASE sites was 2.5-fold greater than the coverage across all positions.

Nineteen DASE sites were mapped to coding regions, with 56% being missense and 41% being synonymous variants. Only one eSNV mapped to the *HLA-C* 3´ UTR. Furthermore, DASE sites are in 13 autosomal genes on eight chromosomes (**Table S2**), with most eSNVs on chromosome 6. The major histocompatibility complex (MHC) locus harbored 24 (70%) of the DASE sites (**Figure 1**). *HLA-B* (n = 10) and *HLA-A* (n = 10) carried the highest number of affected variations, followed by *HLA-C* (n = 4). Only one eSNV changed significantly in the other ten genes *(BRD2, EHD2, GFM2, GSPT1, HAVCR1, MAT2A, NQO2, SUPT6H, TNFRSF11A, and UMPS)*. *HLA-C* harbored DASE sites in all lung cell lines included in this study (**Figure 1**). Both Calu-3 and A549 also shared DASE sites in the *HLA-B* gene. No significant association was observed between the number of DASE sites from the different multiplicity of infection (MOI) ratios and hours post-infection (hpi), suggesting that the mechanisms underlying the differential expression of some alleles may be independent of these variables.

Gene ontology (GO) over-representation analysis revealed that upregulated genes are mainly involved in antigen processing and presentation of endogenous peptides *via* MHC class I (GO:0019885), cell killing (GO:0001906), and regulation of leukocyte-mediated cytotoxicity (GO:0001910) (**Figure S1**). We observed an association between *HLA-A* and *HLA-B* with IFN-γ (GO:0032609) and interleukin-12 production (GO:0032615). *GFM2* and *GSPT1* were associated with the biological process of translational termination (GO:0006415). We also noticed an enrichment of the guanyl ribonucleotide binding (GO:0032561) molecular functions linked to *EHD2*, *GFM2*, and *GSPT1* (**Figure S2**)*. TNFRSF11A* showed significant over-representation in the tumor necrosis factor-activated receptor (GO:0005031) and death receptor (GO:0005035) activities. *HAVCR1* displayed virus receptor activity (GO:0001618), whereas *NQO2* had a function of chloride ion binding (GO:0031404) (**Figure S3**).

### The expression profiles of the genes harboring DASE sites distinguish genetic regulatory mechanisms triggered by infection

**Figure 2:**
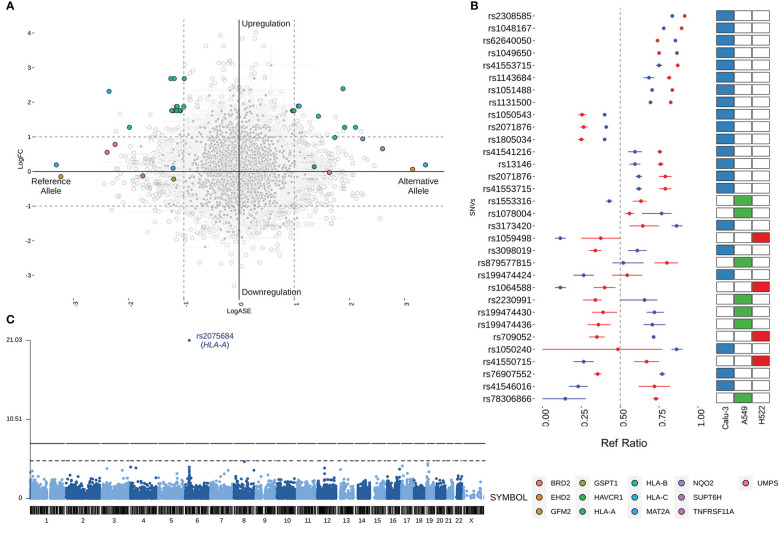
Comparison of the LogASE and LogFC from differential gene expression (DGE). **(A)** Plot showing the LogASE values for DASE sites on the x-axis and LogFC from the DGE comparing the infected with mock-treated cells. Positive LogASE values represent the increase of the alternative allele over the reference. In contrast, negative values represent ASE sites that exhibited a preferential expression of the reference allele after infection. Colored circles show the genes where each DASE site is mapped, whereas gray circles show the eSNVs that met the requirement of FDR > 10% in the DASE analysis. **(B)** Comparison between the Ref Ratio values of SARS-CoV-2 infected and mock-treated cells. The plot shows the ref ratio values on the x-axis and DASE sites on the y-axis. Red and blue circles represent the mean of Ref Ratio values among the replicates of SARS-CoV-2 infected and mock-treated cells, respectively. The interval bars denote the range between the min and max values found across the replicates. Ref Ratio values > 0.5 represent the preferential expression of the reference allele, whereas values < 0.5 show the bias towards the alternative allele. The heatmap at the right highlights the cell line where the DASE sites were found. **(C)** Manhattan plot showing the DASE sites found in iPSC-derived airway epithelium basal cells (iBCs).

The allelic imbalance observed at DASE sites could result from the differential gene expression (DGE) induced by SARS-CoV-2. So, we compared the LogASE values to the log_2_-fold change (LogFC) of the significant DGE (**Figure 2A**). We found that 23 DASE sites were linked to increased expression of the *HLA-A*, *HLA-B*, and *HLA-C* genes at 24 hpi in Calu-3, A459, and H522 cell lines (**Table S2**). That finding showed that HLA expression was increased in a chromosome copy-specific way. Such differentiation was detected across the seven experiments included in our study. For 14 eSNVs in *HLA-A* (n = 7), *HLA-B* (n = 6), and *HLA-C* (n = 1), upregulation was seen in DASE sites where the reference allele was more likely to be expressed (**Figure 2B**). For ten eSNVs in the upregulated group, the expression levels of the alternative allele were higher than the reference allele upon SARS-CoV-2 infection (**Figure 2B**). In Calu-3 cells, the rs713031 in the *HLA-B* gene showed random allele expression over time, with an allelic imbalance towards the alternative allele at 24 hpi with MOI = 10 and switching to the reference allele at 48 hpi with MOI = 0.1. For both experiments, an increased transcriptional level was detected for the gene. Since both comparisons where this eSNV was found came from cells with the same genotype, the transcriptional allele switch may result from a random allelic imbalance.

Twelve genes comprising 13 DASE sites showed compensated expression, which means that the virus did deregulate their expression level. At six eSNVs, the reference allele was expressed more than the alternative allele. However, at seven DASE sites, the alternative allele was expressed more. In this group of genes, there was no change in the way the same eSNV was expressed between different tests. For the rs2071876 in *BRD2*, we identified a consistent expression of the reference allele in the H522 cell line at 72 and 96 hpi (MOI = 0.06).

Furthermore, *HLA-B* and *HLA-C* also displayed compensated gene expression at 12 hpi despite being upregulated at 36 hpi in the same Calu-3 cell (MOI = 10). Though the gene expression changed, for rs41553715 in *HLA-B*, the expression of the alternative allele was increased in both scenarios. Interestingly, the reference allele was preferentially expressed during upregulation of the gene at 48 hpi in Calu-3 (MOI = 0.1) for the same genetic variant, suggesting biased allele expression or a parental-dependent effect. Similar results were found when comparing different cell lines for HLA-C; for the rs41550715, the alternative allele was preferentially expressed regardless of gene expression compensation in A549 or upregulation in Calu-3 (**Table S3**).

### *HLA-A* allele expression is also altered in iPSC-derived airway epithelium basal cells

Next, we aimed to verify the expression profiles of genetic variants across alternative cell lines to determine the extension of the DASE events. We then performed the ASE analysis on a dataset of airway epithelium basal cells derived from iPSC lines (iBCs). The iBCs originated from two independent precursors (iBCs-1566 and iBCs-BU3 NGPT). Unlike lung-derived cell lines, we could not retrieve WES data from both cells. Therefore, the genetic variants identified were considered theoretically heterozygous. We interrogated 26,420 sites, including 14,909 from iBCs-1566 and 16,338 from iBCs-BU3 NGPT. The SNV rs2075684-T-A located in the *HLA-A* gene was found to be differentially expressed during viral infection in iBCs-1566 cells after 24 hpi (**Figure 2C**; **Table S3**). After infection, the expression level of the reference T allele was higher than the alternative A allele. This variation changes phenylalanine to tyrosine at position 33 (Phe33Tyr) of the HLA-A protein. However, the alleles carrying the phenylalanine codon were preferentially expressed. The overall minor allele frequency of the rs2075684 SNV is 0.14 (GnomAD) (49). The expression profiles of two other *HLA-A* variants, rs45585732 and rs1655894, were changed during infection. These SNVs are physically close to each other (interpolated sex-average genetic distance = 6.90515E-05 Kos cM apart; hg19; http://compgen.rutgers.edu/map_interpolator.shtml) (50).

In iBCs (BU3 NGPT), we detected two DASE sites after 72 hpi: rs2269350-G-A (*RPSA*) and rs11724369-G-A (*UVSSA*) (**Table S3**). Both genetic variants had a synonymous functional annotation. The expression of the reference alleles went down, and then the expression of allele A went up. The alternative allele has a relatively elevated frequency across the populations in GnomAD (MAF = 0.26 and 0.29, respectively). We also identified expression perturbations across five neighboring SNVs (rs2276903, rs28614045, rs9996817, rs9685761, and rs6838561) in the *UVSSA* gene. All three genes highlighted in iBCs displayed a compensated gene expression profile in the experiments.

### DASE sites are not related to chromosomal aberrations differences between mock-treated and SARS-CoV-2 infected samples

**Figure 3:**
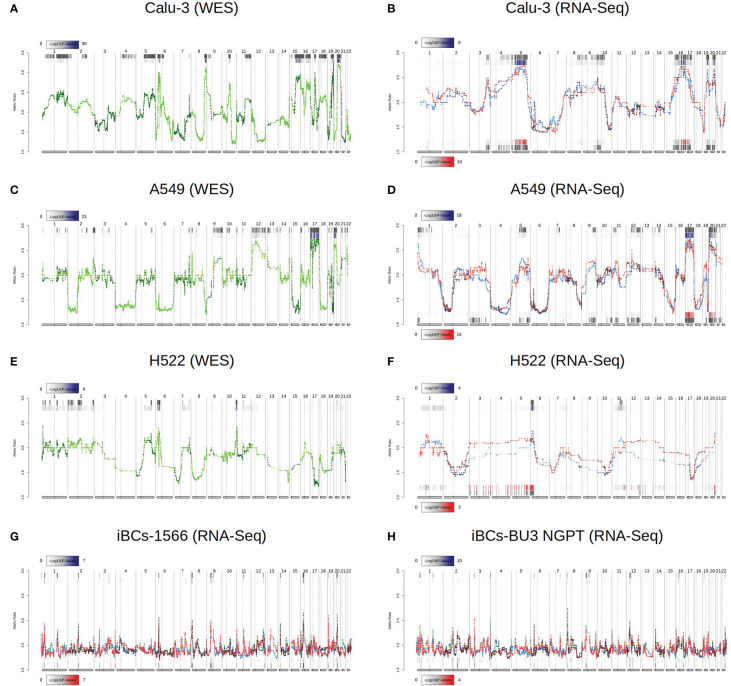
Comparison of chromosomal aberrations between mock-treated and SARS-CoV-2-infected cell lines. Comparison between e-Karyotyping analysis of samples from whole-exome sequencing and RNA-Seq data from Calu-3 **(A, B)**, A549 **(C, D)**, and H522 **(E, F)**. **(G, H)** e-Karyotyping analysis in RNA-Seq data from iPSC-derived airway epithelium basal cells (iBCs) from 1566 and BU3 NGPT cell lines. For each experiment, red dots and lines represent SARS-CoV-2-infected replicates, whereas blue dots and lines show mock-treated replicates. Diploid samples usually display an allelic ratio (y-axis) around 1.4 as previously shown (44, 51). The gray background shown at the top and bottom of each plot shows regions that reach statistical significance for aneuploidy using the piecewise constant fit algorithm. The color gradient displayed next to each region represents the FDR-corrected P value for both comparisons.

Having identified DASE sites across lung-derived and airway basal epithelial cell lines, we asked whether these allele biases were caused by genomic instability or viral infection. We wished to rule out possible karyotype differences as the primary source of ASE since Calu-3, A549, and H522 are hypotriploids (43). We confirmed the chromosomal aberrations in the three cell lines using eSNP karyotyping and WES karyotyping (**Figure 3**). Though the eSNP-Karyotyping revealed a dynamic pattern in the RNA-Seq data of Calu-3, no significant karyotype alterations were detected at the DASE sites (**Figure 3**). For A459, the nine DASE sites identified are mapped at chromosomes 2, 5, 6, and 19, of which six SNVs target the MHC class I locus (**Figure 3**). At the genomic level, we detected significant alterations in chromosomes 17 and 20. Both aberrations were also present at the transcriptional level, as reported by eSNP-Karyotyping analysis in all A549 experiments. The pattern was consistent when both conditions were compared. Karyotyping with WES or RNA-Seq data in H522 cells suggested the presence of a structural aberration across the MHC locus (**Figures 3E, F**). Infected cells have a DASE site in the HLA-C gene. This suggests that the observed allelic shift is related to SARS-CoV-2 infection. We could not retrieve WES data from the iBCs lines used in our study. Despite this, the allelic ratios from RNA-Seq data were consistent in both IBC cell lines, implying that no chromosomal aberrations were present (**Figure 3**). Thus, the DASE sites are not likely to be caused by the alterations in the karyotype of the mock-treated and infected samples.

### Allelic imbalance at DASE sites is partly linked to the differential expression of haplotype blocks

To understand the extension of the allelic imbalance across neighboring eSNVs, we expanded our screening around the DASE sites of each gene. In seven experiments, the *BRD2*, *HLA-C*, *MAT2A*, *RPSA*, *SUPT6H*, and *TNFRSF11A* genes each displayed only one eSNV. Also, even though they had multiple eSNVs, the LogASE values of co-localized variants in four genes *(EHD2, GFM2, GSPT1, and UMPS)* did not change. We then sought to evaluate if these variations overlapped single gene isoforms. By mapping each DASE site and its nearby eSNVs to the transcripts, we saw that all eSNVs were in areas where more than one isoform passed through. Thus, the possibility of isoform-specific allele expression was excluded from this set of genes. Lastly, we saw that neighboring DASE sites were changed after SARS-CoV-2 infection for six genes in 14 experiments. For instance, *HLA-B* displayed many impacted SNVs close to the DASE sites in Calu-3 and A549 cell lines. Similar results were found for the *HAVCR1*, *NQO2*, and *UVSSA* genes.

**Figure 4:**
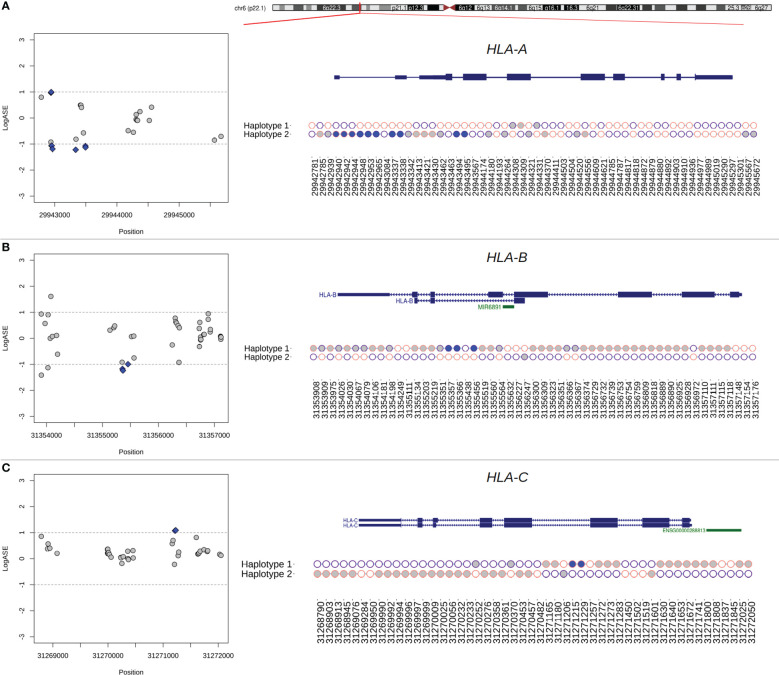
Phasing of DASE sites and co-localized eSNVs from classical MHC class I genes using RNA-seq data from Calu-3 cells. **(A–C)** Regional plot of eSNVs localized around the *HLA-A*, *-B*, and *-C* genes. In blue, DASE sites revealed by the binomial test. The gray represents the other eSNVs tested that did not reach statistical significance. The ideogram of the chromosome is also shown, and a red tick shows where each relevant transcript isoform is located. Next, a plot showing the single haplotype block spanning the genes under analysis. Purple circles represent the reference allele, while the alternative is represented in pink. The x-axis refers to the genomic position of each eSNV in the GRCh38 genome assembly, and the y-axis shows the two haplotypes from the chromosomal locus. Blue and gray circles matched the SNVs in the regional plot on the left. The SNVs found in HLA genes that were not used in the binomial test are shown by the empty circles.

The overwhelming occurrence of eSNVs at the MHC locus raises the question of whether the eSNVs are in phase, i.e., in the same RNA molecule and transcribed from the same parental allele. We use HapTree-X to reconstruct longer-range haplotypes using allelic imbalance at theoretically heterozygous eSNVs (45). We focused our analysis on six genes with DASE sites that span at least two heterozygous SNVs. We reconstructed the phased haplotype for all genes investigated across the different experiments. DASE sites affected by SARS-CoV-2 infection co-localized on the same RNA molecules raising the possibility of viral-induced differential haplotype expression (DHE) (**Figure 4**). This pattern was consistently observed in the *HLA-B* gene throughout seven different comparisons. DHE also occurred in the *HLA-C* and *UVSSA* genes in at least two comparisons.

A single haplotype block spanned the entire *HLA-B* gene, covering a genomic window of 3,268 bp in Calu-3 cells (**Figure 4**). We identified 80 SNVs, of which 44 were interrogated during DASE analysis, five of which were differentially expressed. All the other affected eSNVs that did not reach statistical significance or pass the LogASE threshold were in phase with DASE sites. In the other experiments, the reconstruction of the HLA-B haplotype was fragmented. Still, we found haplotype blocks with DASE sites and co-localized eSNVs where all alleles with different expression patterns during viral infection were phased. Single haplotype blocks were also detected in *HLA-A* and *HLA-C* (**Figure 4**). Ten DASE sites were identified in *HLA-A* in Calu-3 and were co-expressed with the 18 eSNVs in Haplotype 2 (**Figure 4**). For *HLA-C*, we noticed DHE toward haplotype #2, similar to that observed for non-HLA genes such as *HAVCR1* and *UVSSA*.

### DASE sites and affected co-localized SNVs discriminate MHC class I alleles preferentially expressed during infection

The reconstruction of extended haplotype blocks in the MHC class I locus allows allelic typing, which provides insights into the preferential expression of alleles during SARS-CoV-2 antigen presentation. Thus, to predict the HLA alleles assigned to each haplotype reconstructed in the previous analysis, we performed sequence-based HLA typing from RNA-Seq reads in each sample. We identified six samples heterozygous for HLA alleles that displayed DHE, in which the DASE sites and co-localized SNVs could discriminate the HLA allele preferentially expressed (**Table 1**). The *HLA-A* gene of Calu-3 cells was heterozygous for the A*24:02 and A*68:01 alleles. The 10 DASE sites with increased expression after infection mapped to the A*68:01 allele. In the *HLA-C* gene from Calu-3, the DASE sites did not distinguish the heterozygous HLA alleles. Using the SNVs in phase with DASE sites, we could differentiate the imbalance between the two alleles. We found the preferential expression of the allele C*15:02 co-expressed with the allele C*07:02 with three discriminant variations. The DASE sites found in *HLA-B* of Calu-3 did not distinguish alleles. However, by extending our analysis to three neighboring SNVs, we found an imbalance between the alleles B*51:01 and B*07:02. We observed that two altered SNVs mapped to B*51:01 whereas B*07:02 was characterized by a single variant (**Table 1**). Lastly, we found that the B*44:03 and B*18:01 alleles of the *HLA-B* gene were both heterozygous in the A459 experiments.

**Table 1:** DASE sites and affected co-localized SNVs in MHC class I alleles.

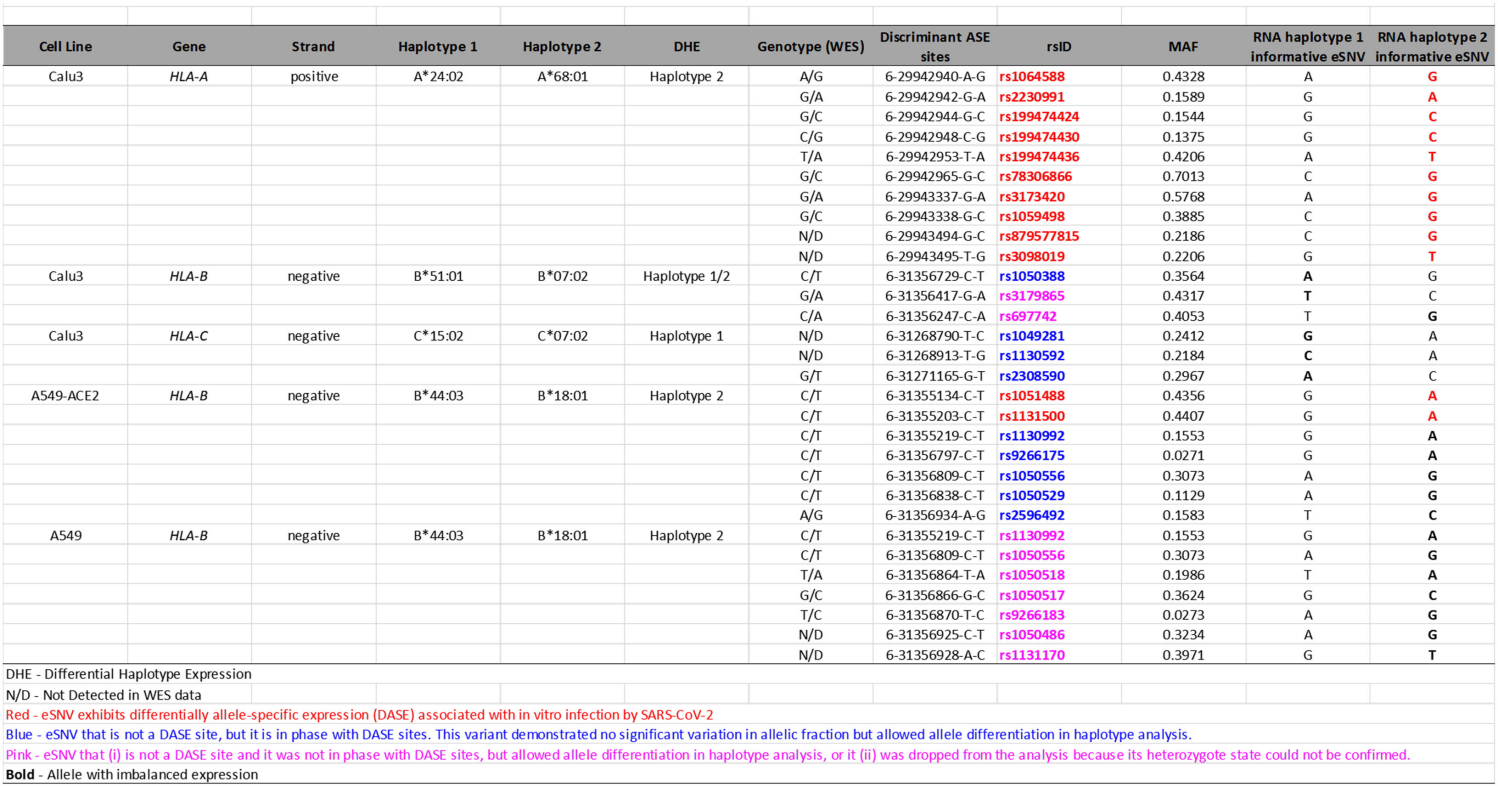

We tracked the informative DASE sites over time to describe the flux differences of HLA haplotype expression during the infection. Allele flux over time can be measured if there are more than one DASE site on each HLA locus at different time points and/or MOIs and if the identified DASE site(s) distinguish(es) the HLA haplotypes. For *HLA-A* in Calu-3, the ten DASE sites investigated suggested a continuous expression of the allele with no changes in the preferential expression of the A*68:01 allele over time. For *HLA-B* of A549-ACE2 cells, we observed a constant expression of B*18:01 across different MOIs. The continual differential expression of rs1051488 and rs1131500 SNVs determined the allelic flux. We could not determine the allelic flux for *HLA-C* alleles because the DASE sites did not differentiate the haplotypes. In H522 cells, a single DASE site was identified on *HLA-C* at 96hpi and MOI 0.25, which did not allow the determination of the allelic flux.

## Discussion

This study identified an imbalanced expression of genetic variations in classical MHC class I genes and ten other genes associated with SARS-CoV-2 infection. Gene ontology analysis showed that the 13 genes with DASE sites in Calu-3, A549, and H522 are enriched in protein binding functions, some of which are involved in SARS-CoV-2 infection, COVID-19 disease progression, and severity. We included ACE2 receptor positive (Calu-3) and negative but permissive to SARS-CoV-2 infection (A549 and H522) cell lines. The Calu-3 cell line is airway epithelial derived from human bronchial submucosal glands (52, 53). The A549 cell line recapitulates features of the phenotype of the multifunctional alveolar type II (ATII) epithelial pneumocytes, capable of surfactant production and expression of high numbers of multilamellar bodies (54, 55). ATII pneumocytes are essential in regenerating the alveolar epithelium following lung injury and thus contribute to lung defense. The H522 cell line is also airway epithelial but permissive to infection by SARS-CoV-2 (13).

Interestingly, most DASE sites were found in Calu-3. The difference may be linked to the extent to which the cells are permissive to virus entry, replication, and the differential expression of the ACE2 receptor. Transcriptome analyses of the lower lung are mostly limited to ATII pneumocytes (56). However, a variable infection gradient has been observed in the upper and lower respiratory tract, which parallels the gradient of ACE2 expression (57). We noted that the HLA allele shift is neither cell line type restricted nor ACE2-dependent because the virus-induced HLA allele-switching was observed in cell lines discordant for ACE2 receptor expression. The HLA allele switch is expected to occur in other cell lines, being used to refine antigen recognition during injury. Whether the observed HLA allele switch occurs during a non-infectious cell injury is unclear.

*HLA-A*, *-B*, and *-C* genes act on endogenous peptide antigen presentation and are associated with disease susceptibility. The transcriptional regulator bromodomain-containing protein 2 (BRD2) is a potent regulator of ACE2 transcription in Calu-3 cells (58). The EHD2 protein, highly enriched at the neck of caveolae, controls a cell-autonomous, caveolae-dependent fatty acid uptake pathway by adipocytes, endothelial cells, and muscle cells (59). Importantly, *EHD2* is underexpressed in obese patients, a known risk comorbidity for severe COVID-19. COVID-19 is less likely to happen in people with the cytosolic glutathione S-transferase GSPT1 rs1695 allele (60). The hepatitis A virus cellular receptor (HAVCR1, also called KIM1), used by Ebola, Marburg, Dengue, and Zika viruses, is an entry factor for SARS-CoV-2 to kidney cells, where the virus induces organ abnormalities associated with poor prognosis and mortality in COVID-19 patients (61). The methionine adenosyltransferase 2A (*MAT2A*), involved in S-adenosylmethionine methylation pathways, is differentially upregulated in mono-CD14+CD16+ cells in patients with severe COVID-19 (62). MAT2A presumably is required to methylate the SARS-CoV-2 RNA cap structures, allowing genome transcription and preventing the recognition of RNA Cap structures by cellular innate immunity receptors (63). The *SUPT6H* gene codes one of the many RNA-binding proteins profoundly down-regulated upon SARS-CoV-2 infection (64). The uridine monophosphate synthase (UMPS) is involved in pyrimidine biosynthesis, and pyrimidine inhibitors synergize with nucleoside analogs to block SARS-CoV-2 replication (65).

The observed allele bias in classical MHC class I genes leads to the preferential expression of one allele within a heterozygous locus, showing that the upregulation of these genes is driven in a haplotype-specific manner. The classical MHC class I molecules handle mainly self-peptides or viral antigens. The exposure of the HLA-peptide complex on the cell surface is followed by CD8+ cytotoxic T lymphocyte binding, which may induce apoptosis in virally infected cells and generate long-term immunological memory. With heterozygous variant sites in the HLA-A, -B, and -C genes, each cell can express up to six MHC class I alleles simultaneously. Perturbations in MHC allelic expression can change how antigens are presented. The cellular immunity conferred by CD8+ memory T cells is crucial to fighting the earlier SARS-CoV-1 infection and the current SARS-CoV-2 pandemic, even with or without humoral responses (66–69). Though our findings are limited to the repertoire of antigens for T CD8+ cell presentation, the isoform expressed may play a role in the efficiency of the immune response to viral infection.

For example, the A*68:01 allele overexpressed in Calu-3 has been predicted to have a high binding affinity to SARS-CoV-2 epitopes (70). A*68:01 is a common allele found across different populations at 5.2-25% frequency. This allele was strongly associated with mortality from influenza A (H1N1) infection (71, 72). A large-scale analysis also revealed a proclivity for the worst COVID-19 outcome in patients with the B*51:01 allele that is overexpressed in Calu-3-cells (73). *In silico* analysis identified a high affinity for potential T-cell epitopes of S-protein (74). Previous studies reported a protective role of B*51:01 in the long-term control of AIDS progression in HIV-infected individuals (75–77). The alternative allele B*07:02, co-expressed with B*51:01, had a beneficial association with high antiviral efficacy against SARS-CoV-2 (78). For *HLA-B* of Calu-3 cells, we were not able to determine the phase of DASE sites considering the two alleles B*51:01 and B*07:02.

**Figure 5:**
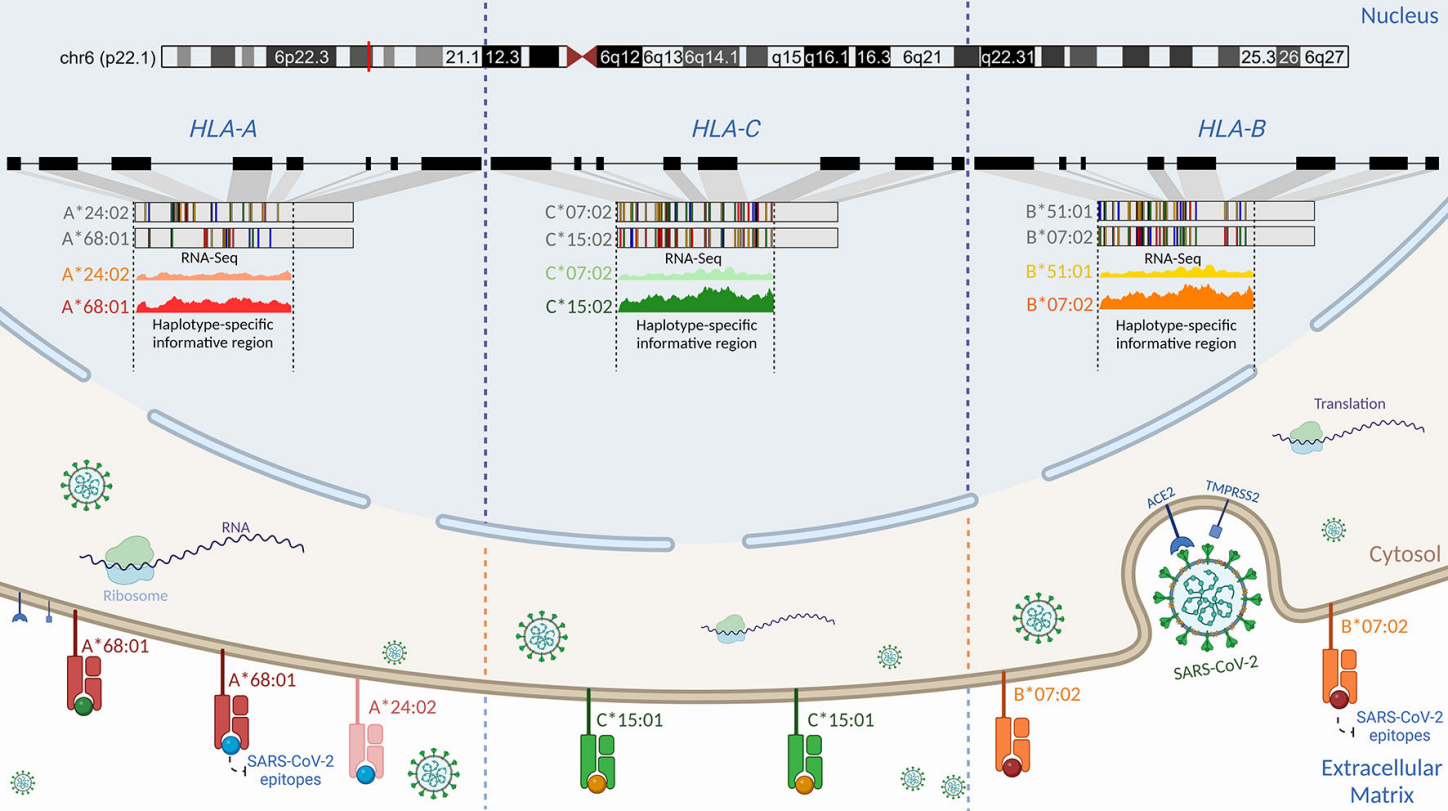
Schematic representation of the proposed regulatory genetic mechanism associated with the haplotype-specific expression of class I HLA alleles during SARS-CoV-2 infection. Viral cell entry triggers preferential transcription of the RNA molecules in the classical class I HLA locus. Even though Calu-3 is heterozygous for *HLA-A*, *-B*, and *-C* alleles (79), we found that the A*68:01 allele was more expressed than the A*24:02 allele, the B*07:02 allele was more expressed than the B*51:01 allele, and the C*15:02 allele was more expressed than the C*07:02 allele. Such differences in the expression may be attributed to structural differences in promoter motifs (80), transcriptional factors, genetic variations, and environment (81, 82). Cross-referencing analysis using HLA peptidome data from Calu-3 infected by SARS-CoV-2 generated by Nagler and colleagues (2021) revealed that the same RNA molecule found to be preferentially expressed in RNA-Seq data corresponds to the HLA protein expressed on the cell surface for classical class I alleles. Nagler and colleagues (2021) reported that most peptides presented on the cell surface matched the A*68:01 allele when compared to the A*24:02. For *HLA-C*, no peptide matching C*07:02 was found after infection. Similarly, the B*07:02 allele was found to be preferentially expressed at the HLA-B locus. By using DASE, swapping alleles with low binding affinity could be a part of the defense that helps COVID-19 outcomes be less severe. Created with BioRender.com.

Cross-referencing with the HLA peptidome in Calu-3 infected by SARS-CoV-2 revealed that the epitopes on the cell surface matched most of the HLA alleles that were differentially expressed in our analysis (79). The majority of peptides presented by HLA-A on the Calu-3 surface matched the A*68:01 allele. Nagler and his team did not see B*51:01 and C*07:02 being expressed in SARS-CoV-2-infected Calu-3. These results help settle the disagreement about which *HLA-B* haplotype is most strongly expressed at the RNA level. Thus, the absence of epitopes matching the alternative allele for both HLA-*B* and *HLA-C* genes shows that the differential haplotypic expression may be reflected at the protein level. It is still not clear if the immunodominant epitope controls the preferential expression of the HLA alleles or if the different expression of the HLA alleles makes some peptides more likely to be chosen. The three HLA alleles upregulated in Calu-3 may play a protective role against COVID-19 (**Figure 5**). A*68:01 showed a protective effect against severe manifestations of the disease in Tapachula-Chiapas, Mexico (83). In contrast, the peptides presented by A*68:01 derived from the envelope protein are homologous to the neuronal cell adhesion molecule (NCAM) (84). A*68 has been associated with developing Guillain-Barré syndrome (GBS). B*07:02 and C*15:02 have antiviral activity and resistance against SARS-CoV-2, respectively (78, 85).

In the *HLA-B* gene in A549 cells, we observed the preferential expression of the B*18:01 allele over the B*44:03 allele in all experiments. B*18:01 was associated with the manifestation of subacute thyroiditis triggered during the SARS-CoV-2 infection (86). This allele has also been linked to T cell cross-reactivity between EBV epitopes and a self-peptide, causing an aberrant immune response (87). The HIV viral replicative capacity was significantly higher in subjects expressing the B*18:01 allele (88). In contrast to the patterns seen with *HLA-A* and *-B*, there is no clear link between the allele C*15:02 and COVID-19. This allele was upregulated in Calu-3 experiments when co-expressed with C*07:02. The C*15:02 allele confers resistance against SARS-CoV infection (85). Francis and colleagues (89) recently described the HLA-B*07:02 allele presenting homologous epitopes from SARS-CoV-2 and other human coronavirus, providing high pre-existing immunity. Preferential expression of HLA alleles may be closely connected to TCR repertoire diversity (89). Moreover, HLA genotypes and CD8+ T cell responses have been described as having implications for herd immunity and strategies to consider during vaccine design to warrant long-term immunity against SARS-CoV-2 (89, 90).

Zhang et al. reported allelic imbalances across HLA-B alleles in lung cell lines infected by SARS-CoV-2 using an alternative methodological approach. The authors offered three non-exclusive biologically plausible mechanisms to explain the differential haplotype expression: (i) the activation/silencing of one allele is attributed to pathological effects, (ii) independent regulation of the transcription of both alleles, and (iii) the presence of cis-acting regulatory elements (27). In our study, the occurrence of DASE sites in the *BRD2* gene mapping to the HLA chromosomal region corroborates the cis-acting regulatory elements’ hypothesis. During T cell activation, allele-specific expression changes were described in HLA and other autoimmune loci for CD4+ T cells (91).

### Limitations and strengths

We warn against drawing any actionable, functional conclusions from these results because the sample size was small, and the study was done with secondary WES and RNA-Seq public data. An important caveat of the study is the limited number of informative DASE sites in HLA genes that are required to analyze HLA-allele differences over time. The number of informative DASE sites depends on the heterozygous status of contiguous SNVs, therefore, on the genetic background of the sample donor. The determination of allele flux over time depends on (i) the HLA alleles being typed in the cell line, (ii) more than one DASE site occurring on the HLA genes in the different MOIs experiments, (iii) the identified DASE sites discriminating HLA haplotypes. All that granted, the flux of HLA alleles could be estimated for the HLA-A gene in Calu-3 and HLA-B in A549-ACE2 transfectant cells.

ASE perturbations are not mechanistically unique to SARS-CoV-2 infection, despite the reported shift in allele expression in HLA and ten other genes. Multiple ASE alterations have been identified in CD4-T cells infected by the oncogenic Marek’s Disease herpesvirus (MDV) (92). MDV caused ASE changes in six genetic resistance loci (*MCL1, SLC43A2, PDE3B, ADAM33, BLB1*, and *DMB2*) that are related to T-cell activation, T-cell and B-cell receptors, ERK/MAPK, and PI3K/AKT-mTOR signaling pathways, all of which play important roles in MDV infection. Because ASE-affected genes represent the complex trait of genetic resistance to Marek’s disease, the trait is then determined by transcriptional regulation (93). Our results show that when SARS-CoV-2 infects cells, there is a transcriptional allelic flip in the affected genes, which occurs regardless of compensation of gene expression. We hypothesize that when the virus enters the cell, a DASE flip regulatory mechanism swaps HLA alleles that display epitopes with poor binding affinity. Functional studies are required to assess the biological significance of the transcriptional allelic flip. Whether the transcriptional allelic flip induced by SARS-CoV-2 infection is a transient or long-lasting phenotype will demand transcriptome and proteome comparative analysis in cultured cells cured of SARS-CoV-2. The biological significance of the transcriptional allelic flip is for the virus-induced selection of HLA alleles with higher affinity. Testing that hypothesis will require measuring differential peptide recognition in infected cells.

## Data availability statement

The original contributions presented in the study are included in the article/**Supplementary Material**. Further inquiries can be directed to the corresponding authors.

## Author contributions

RSFJ, EMA, and ATRV contributed to conception and design of the study. RSFJ, CSF and YM conducted the bioinformatic analyses. RSFJ performed the statistical analysis. RSFJ and EMA wrote the first draft of the manuscript and prepared figures and tables. RSFJ, TMLS, JRT, EMA, and ATRV wrote sections of the manuscript. All authors contributed to manuscript revision, read, and approved the submitted version.

## Supplementary material

Supplementary Table 1 Public RNA-Seq and WES experiments included in this study.

Supplementary Table 2 DASE sites identified in Calu-3, A549, and H522 cell lines.

Supplementary Table 3 DASE sites found in airway epithelium basal cells derived from iPSCs.
