# Supplementary material for: Differential haplotype expression in class I MHC genes during SARS-CoV-2 infection of human lung cell lines
Source: Front Immunol. 2023 Feb 1;13:1101526. doi: 10.3389/fimmu.2022.1101526 (PMC9929942; doi:10.3389/fimmu.2022.1101526)
Supplement: Supplementary file 2 [file DataSheet_2.pdf]

Supplementary material

Differential haplotype expression in class I MHC genes during SARS-CoV-2 infection of human lung cell lines

Ronaldo da Silva Francisco Junior[1], Jairo R. Temerozo[2,3], Cristina dos Santos Ferreira[1], Yasmmin Martins[4], Thiago Moreno L. Souza[5,6], Enrique Medina-Acosta[7,*], Ana Tereza Ribeiro de Vasconcelos[1,*]

[1] Bioinformatics Laboratory (LABINFO), National Laboratory of Scientific Computation (LNCC/MCTIC), Petrópolis, Brazil

[2] Laboratory on Thymus Research, Oswaldo Cruz Institute (Fiocruz), Rio de Janeiro, Brazil

[3] National Institute of Science and Technology on Neuroimmunomodulation, Rio de Janeiro, Brazil

[4] Instituto de Cálculo, Facultad de Ciencias Exactas y Naturales, Universidad de Buenos Aires (FCEyN-UBA), Buenos Aires, Argentina

[5] Laboratory of Immunopharmacology, Oswaldo Cruz Institute (IOC), Oswaldo Cruz Foundation (Fiocruz), Rio de Janeiro, Brazil

[6] Center for Technological Development in Health (CDTS), National Institute for Science and Technology on Innovation on Neglected Diseases Neglected Populations (INCT/IDNP), Oswaldo Cruz Foundation (Fiocruz), Rio de Janeiro, Brazil

[7] Molecular Identification and Diagnostics Unit (NUDIM), Laboratory of Biotechnology, Center for Biosciences and Biotechnology, Universidade Estadual do Norte Fluminense Darcy Ribeiro (UENF), Campos dos Goytacazes, Brazil

***Corresponding author:**

Enrique Medina-Acosta

e-mail: quique@uenf.br

Ana Tereza Ribeiro de Vasconcelos

e-mail: atrv@lncc.br.

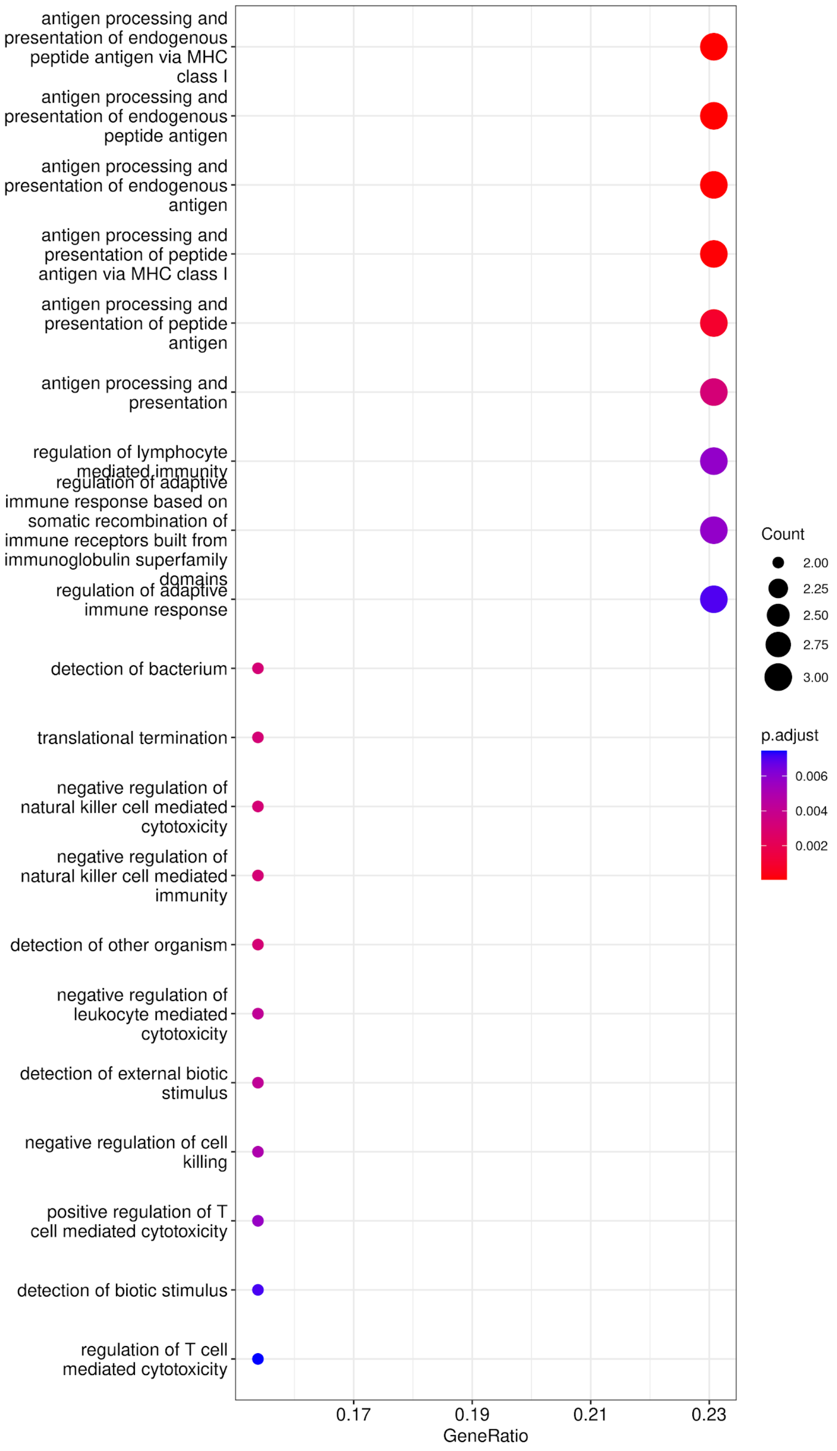

Supplementary Figure 1. Biological processes enriched across the set of 13 genes harboring DASE sites. Ontology analyses using the enrichGO function of the clusterProfiler package in R considering a pvalueCutoff = 0.01 and qvalueCutoff = 0.05 (1,2). The plot shows the top 20 biological process categories enriched within the 13 genes that harbored DASE sites. The X-axis indicates the ratio of genes involved in the biological processes over the total number of genes queried.

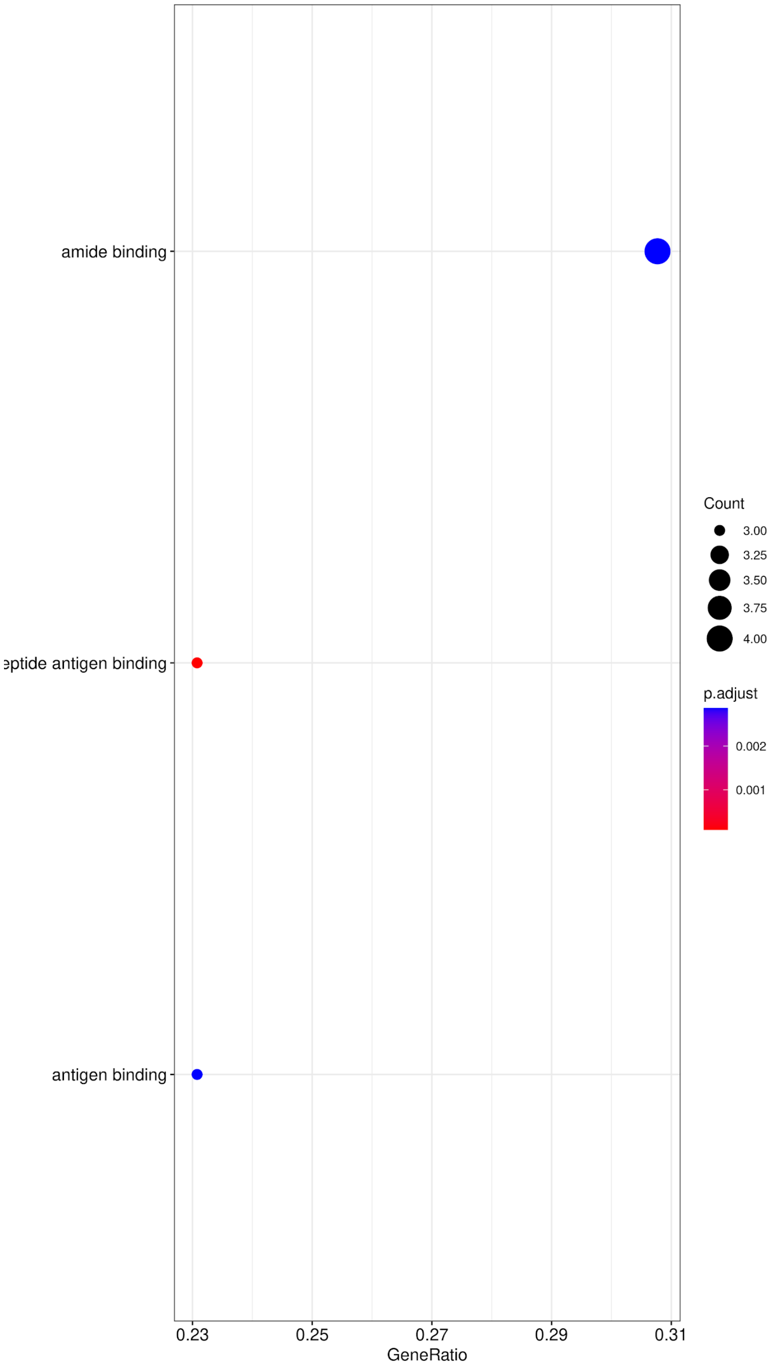

Supplementary Figure 2. Molecular function ontology enriched in a set of 13 genes harboring DASE sites. Ontology analyses using the enrichGO function of the clusterProfiler package in R considering a pvalueCutoff = 0.01 and qvalueCutoff = 0.05 (1,2). The plot shows the most relevant molecular function categories enriched within the 13 genes that harbored DASE sites. The X-axis indicates the ratio of the set of genes involved in each category over the total number of genes queried.

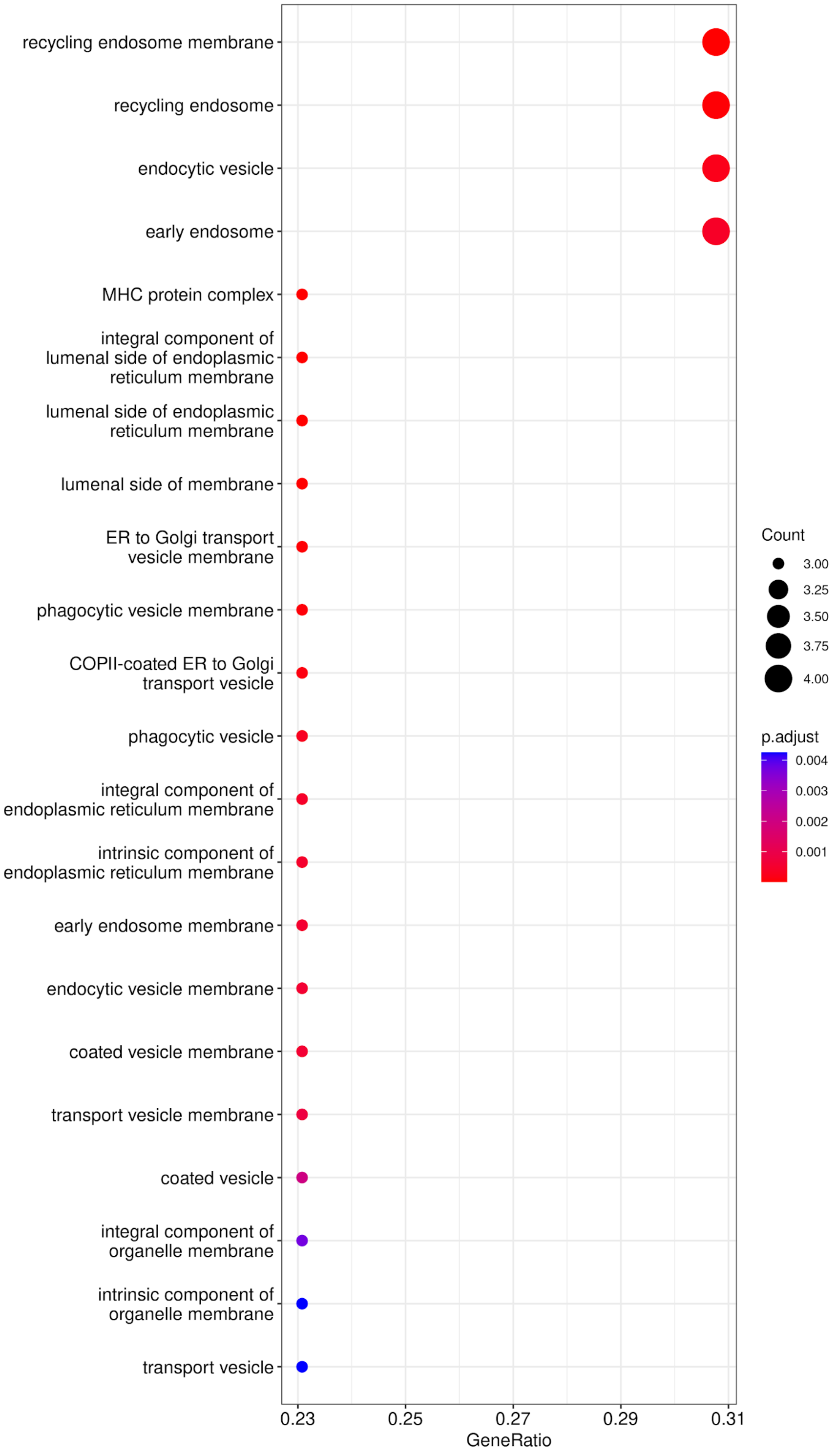

Supplementary Figure 3. Cellular components enriched in a set of 13 genes harboring DASE sites. Ontology analyses using the enrichGO function of the clusterProfiler package in R considering a pvalueCutoff = 0.01 and qvalueCutoff = 0.05 (1,2). The plot shows the top 30 most relevant cellular component categories enriched within the 13 genes that harbored DASE

sites. The X-axis indicates the ratio of the set of genes involved in each category over the total number of genes queried.

References

1. Wu T, Hu E, Xu S, Chen M, Guo P, Dai Z, Feng T, Zhou L, Tang W, Zhan L, et al. clusterProfiler 4.0: A universal enrichment tool for interpreting omics data. *Innovation (Camb)* (2021) 2:100141.

2. Yu G, Wang L-G, Han Y, He Q-Y. clusterProfiler: an R package for comparing biological themes among gene clusters. *OMICS* (2012) 16:284–287.
